# Disproportionately large communicating fourth ventricle: two case reports

**DOI:** 10.1186/s13256-019-2158-9

**Published:** 2019-07-21

**Authors:** Jian Lyu, Ju-bo Wang, Yu Quan, Shouping Gong

**Affiliations:** Neurosurgical Department, Second Affiliated Hospital, Xi’an Jiaotong University, Xi’an, 710004 China

**Keywords:** Hydrocephalus, Fourth ventricle, Syringomyelia, Fourth ventriculomegaly

## Abstract

**Background:**

Management of the disproportionately large communicating fourth ventricle is still problematic.

**Case presentation:**

Two cases of disproportionately large communicating fourth ventricle were treated successfully. One was a case of a 51-year-old Han Chinese woman with a complaint of headache and dizziness of 1 year’s duration. Magnetic resonance imaging (MRI) demonstrated hydrocephalus with a disproportionately large fourth ventricle. She underwent a ventriculo-peritoneal shunt of the right lateral ventricle. Her symptoms were relieved totally. Five years later, magnetic resonance imaging showed she had a normal ventricular system. The other case was a 24-year-old Han Chinese man with a 2-month history of headache and dizziness accompanied by progressive loss of bilateral vision. Magnetic resonance imaging revealed hydrocephalus with a disproportionately large fourth ventricle, crowded posterior cranial fossa, and syringomyelia extending from C1 to C5. He underwent suboccipital and C1 decompression and duraplasty. Shortly after the surgery, his symptoms were relieved completely, the syringomyelia completely disappeared, and the fourth ventricle became significantly smaller.

**Conclusions:**

The management of the disproportionately large communicating fourth ventricle should be individualized. If it coexists with crowded posterior cranial fossa or syringomyelia, posterior fossa decompression could be an option for initial management. If there is no sign of crowded posterior cranial fossa or syringomyelia, shunt of the lateral ventricles might be the first choice.

## Background

Disproportionately large communicating fourth ventricle (DLCFV) is rare [1]. It was first proposed in 1980 as a subtype of communicating hydrocephalus presenting with a universal enlarged ventricular system including a disproportionately dilated fourth ventricle without history of ventriculo-peritoneal (VP) shunt [2, 3]. The communicating hydrocephalus is usually featured as proportionate enlargement of the lateral, third, and fourth ventricles. However, DLCFV has a fourth ventricle that has dilated to a greater extent than the lateral and third ventricles. It is prone to be confused with the isolated fourth ventricle (IFV), which may lead to a puzzle of management. IFV, which has also been named as trapped or encysted fourth ventricle, is defined as a remarkable dilation of the fourth ventricle due to the obstruction of both the Sylvius aqueduct and the foramina of Magendie and Luschka. However, DLCFV has been limited to the dilated fourth ventricle with patent cerebrospinal fluid (CSF) flow through the Sylvius aqueduct and the foramina of Magendie and Luschka, which is truly communicating hydrocephalus.

The treatment of DLCFV is still problematic because of the poor understanding of its etiology and pathophysiology [4]. Many patients need to undergo multiple shunt revisions or require additional procedures prior to symptom resolution [5]. We report two patients with DLCFV treated with CSF shunt and posterior fossa decompression, respectively.

## Case presentation

### Patient 1

**Fig. 1:**
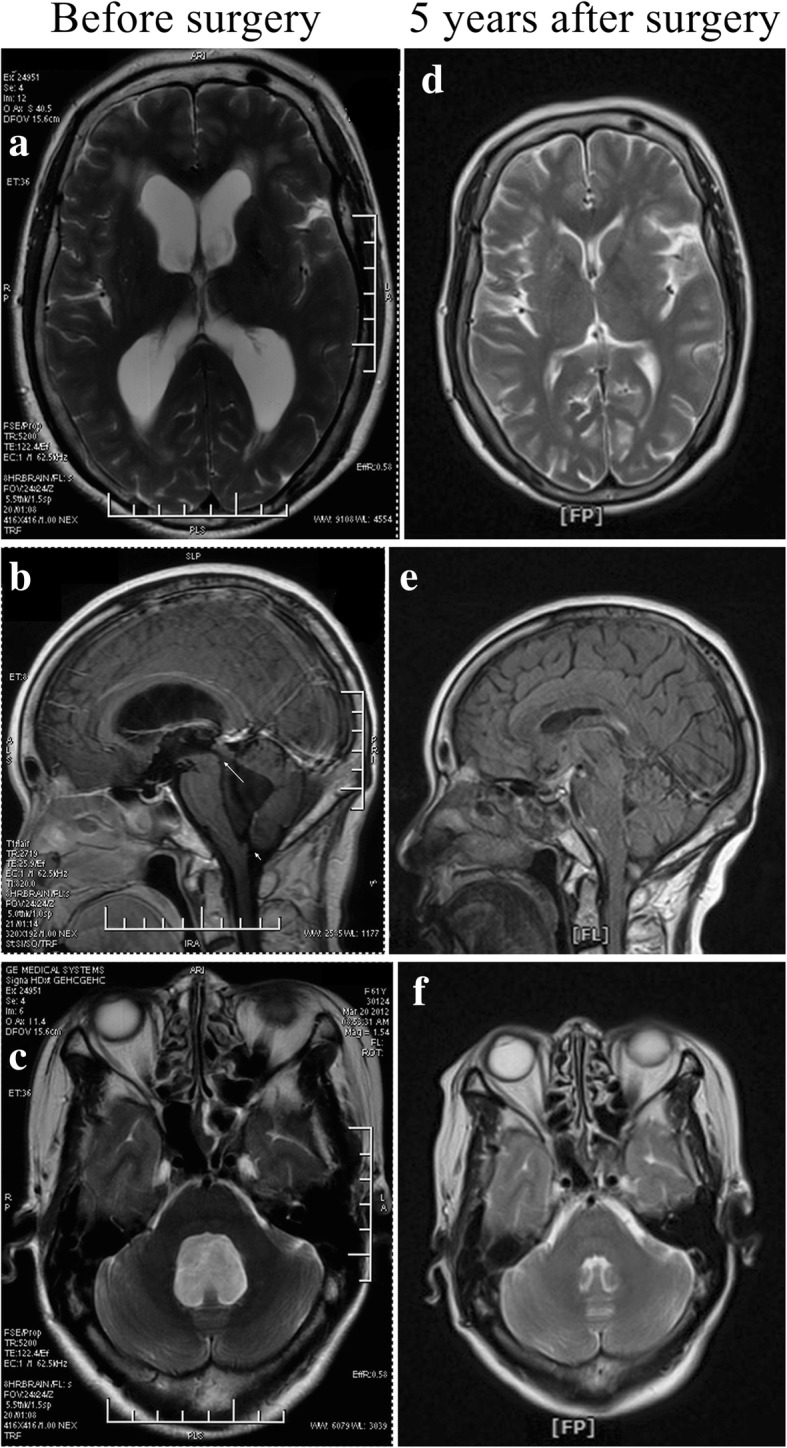
Preoperative (**a–c**) and postoperative (**d–f**) magnetic resonance imaging of a typical disproportionately large communicating fourth ventricle in patient 1. → Preoperative dilated aqueduct

A 51-year-old Han Chinese woman was admitted with a 1-year history of headache and dizziness. She had received no past interventions. She had no medical, family, or psychosocial history. Clinical examination showed the Romberg’s sign. Magnetic resonance imaging (MRI) demonstrated hydrocephalus with a disproportionately large fourth ventricle (Fig. 1a–c). She underwent a VP shunt (PS Medical programmable valve set at 1.0, which was equivalent to the opening pressure of 50–70 cmH_2_O in the upright position; Medtronic, Minneapolis, MN, USA) of the right lateral ventricle and had an uneventful postoperative course. Her symptoms were relieved totally. Five years later, MRI still showed a normal ventricular system (Fig. 1d–f). The parameter of the opening pressure of the programmable valve has never been adjusted because she has never had any discomfort.

### Patient 2

**Fig. 2:**
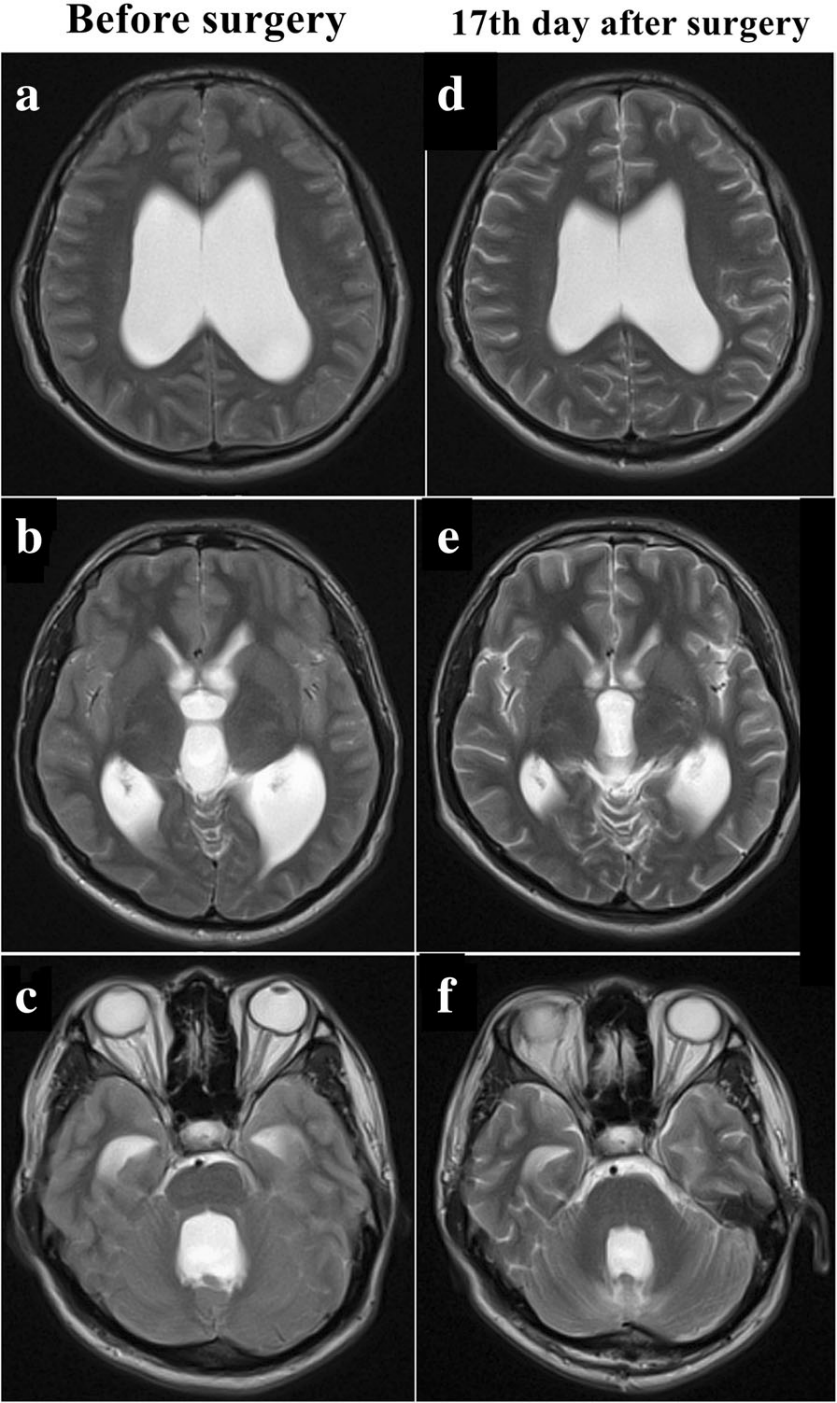
Disproportionately large communicating fourth ventricle coexisting with syringomyelia in patient 2. **a** and **d** Axial images of the lateral ventricle. **b** and **e** Axial images of the third ventricle. **c** and **f** Axial images of the fourth ventricle

**Fig. 3:**
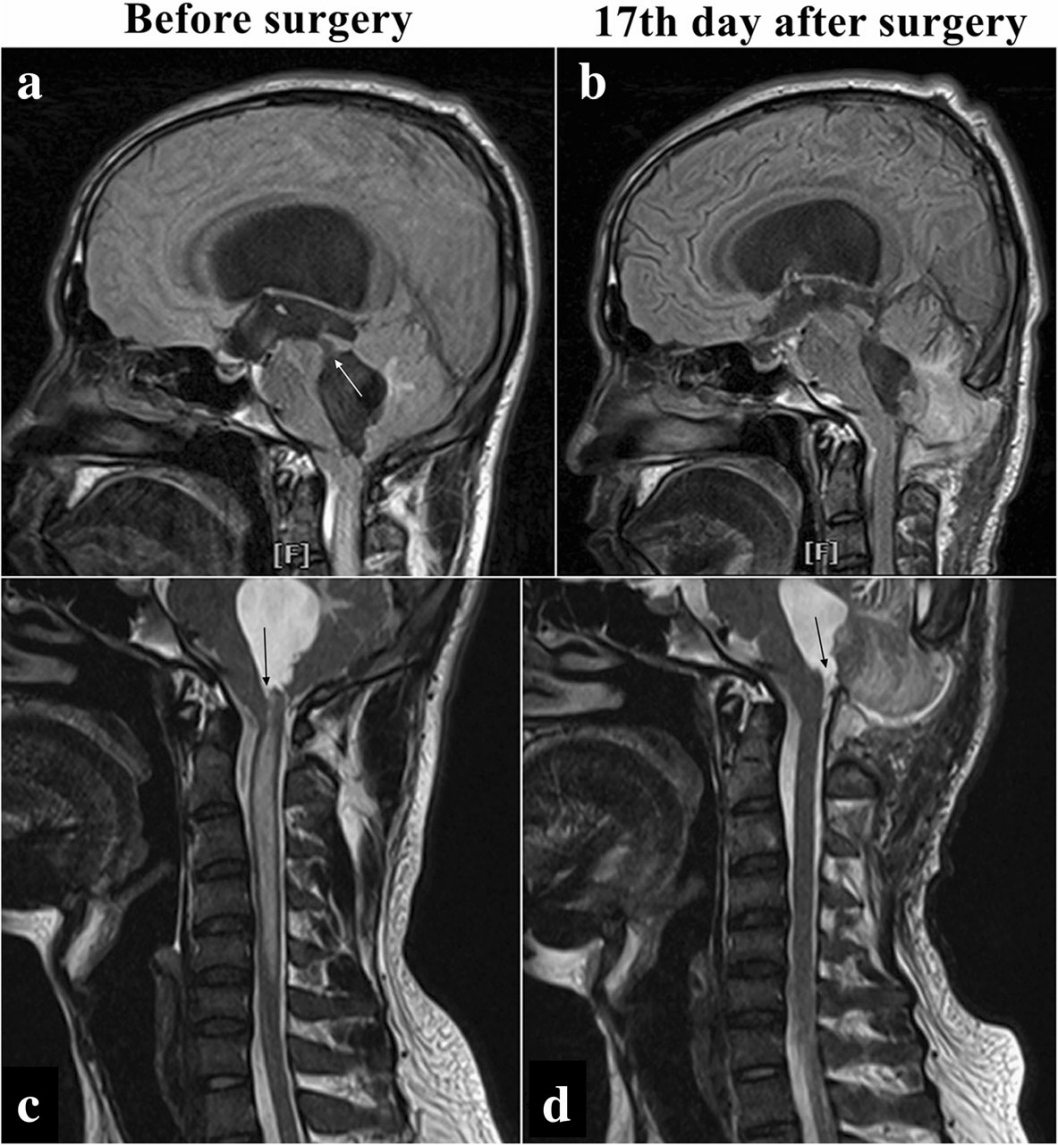
Disproportionately large communicating fourth ventricle coexisting with syringomyelia in patient 2. **a-c** Sagittal images of the fourth ventricle. **c–d** Sagittal images of syringomyelia. *White arrow* points to the dilated aqueduct (**a**); *black arrow* indicates the dilated central canal of the spinal cord communicating with the fourth ventricle (**c**); and *black arrow* points to the reopened outlet of the fourth ventricle (**d**)

A 24-year-old Han Chinese man presented with a 2-month history of headache and dizziness accompanied by progressive loss of vision in both eyes. His symptoms worsened after a cervical massage 1 week before admission. He had frequent paroxysmal headache associated with nausea, vomiting, and blurred vision. He had a history of mumps and viral encephalitis at the age of 4, which had no sequelae. He had received no past interventions. He had no other medical, family, or psychosocial history. On admission, his visual acuity was 0.5 in both eyes. Physical examination showed bilateral severe optic papilledema, forced head position, and bilateral Babinski’s signs. MRI (Figs. 2 and 3) revealed hydrocephalus with a remarkably enlarged fourth ventricle, crowded posterior fossa, and syringomyelia extending from C1 to C5. His Evans index was 0.4 (61.30/152.9). He underwent a suboccipital and C1 decompression and duraplasty. After the operation, his headache and dizziness were relieved rapidly, and both Babinski’s signs disappeared. On the 17th postoperative day, his visual acuity reached 1.2 in the right eye and 0.6 in the left eye, and his bilateral optic papilledema was reduced. MRI (Figs. 2 and 3) showed that the fourth ventricle had become smaller, the trumpet-like aqueduct had become tubular, and the syringomyelia had dramatically disappeared. His Evans index dropped to 0.36 (55.16/151.96). At his 20th-week follow-up, his visual acuity had reached 1.5 in the right eye and 1.2 in the left eye. At his tenth-month follow-up, his vision in both eyes had reached 1.5, and the volume of the ventricular system had further decreased on MRI. His Evans index had dropped to 0.34 (51.5/149.3). He had no discomfort.

## Discussion

Both DLCFV and IFV are characterized by the disproportionately enlarged fourth ventricle. DLCFV is a special type of communicating hydrocephalus, whereas IFV is a special type of obstructive hydrocephalus.

### DLCFV and IFV should be differentiated

As Scotti *et al*. proposed, DLCFV could return to normal after shunting of the lateral ventricles [3]. They stressed that the term *isolated fourth ventricle* should be used only for patients with previous CSF shunt. However, IFV without history of CSF shunt is common [6]. Hawkins stressed that IFV was associated with small lateral ventricles [7]. However, it is not uncommon in the literature that IFV includes the disproportionately enlarged fourth ventricle coexisting with normal-sized or enlarged third and lateral ventricles [2, 6, 8–11]. Qi and Matsumoto proposed the concept of functional obstruction of aqueduct [12]. They divided the obstruction of the aqueduct into two types: functional and permanent. The functionally obstructed aqueduct could reopen when the elevated infratentorial pressure was decreased or overdrainage of the supratentorial shunt system was corrected, which was named *Raimondi’s phenomenon*, but permanent obstruction of the aqueduct required shunt of the fourth ventricle. O’Hare *et al.* stressed that the aqueduct was patent, whereas the foramina of Luschka and Magendie were occluded in the condition of DLCFV [13]. Ogiwara and Morota proposed the concept of “pre-isolated fourth ventricle,” which refers to the occlusion of the fourth-ventricle outlets and the enlargement of the fourth ventricle while the aqueduct is still patent [11]. Ferrer and de Notaris used the concept of functionally trapped or isolated fourth ventricle to define tetraventricular hydrocephalus in association with disproportionate enlargement of the fourth ventricle [4].

We agree to the statement on the functional occlusion of the aqueduct proposed by Qi *et al.* [12, 14]. In conditions of DLCFV, the inlet and the outlet of the fourth ventricle may both be functionally occluded. The disproportionately large fourth ventricle includes two different conditions: DLCFV and IFV. DLCFV is the disproportionately large fourth ventricle communicating with the rest of the ventricular system and subarachnoid space, which is associated with a patent or reversibly occluded Sylvius aqueduct and enlargement of the rest of the ventricular system. IFV is the disproportionately large fourth ventricle, which is permanently isolated from the rest of the ventricular system and subarachnoid space. We do not consider the size of the other ventricles as a criterion to differentiate IFV from DLCFV, because the enlargement of the rest of the ventricular system is a matter of time due to the occlusion of the aqueduct in the condition of IFV without prior supratentorial CSF shunt. The shunt-related IFV in association with small or slitlike lateral ventricles is a particular type of IFV. For IFV, shunting CSF from the fourth ventricle or surgical reopening of outlet or inlet of the fourth ventricle should be the treatment option. DLCFV may progress to IFV if there appears to be a pressure gradient between the supratentorial and infratentorial compartments after the supratentorial shunting.

The etiology and pathophysiological mechanism of DLCFV are still inconclusive. Raimondi proposed that the pressure gradient between supratentorial and infratentorial compartments could lead to the occlusion of the aqueduct when the pressure within the posterior fossa is increasing [15]. It was also Raimondi who described the reversibility of aqueductal stenosis. Qi and Matsumoto observed the reopening of the aqueduct in cases of IFV by upgrading the shunt pressure [14]. Barami *et al.* reported that subzero drainage of CSF from the lateral ventricle could treat fourth ventriculomegaly successfully, which questioned the hypothesis that overshunting of the lateral ventricles leads to functional aqueductal obstruction [16]. However, we believe that the effectiveness of subzero CSF shunting was based on the patency or reopening of the aqueduct, which proved the possibility of the functional occlusion of the aqueduct. For patients with DLCFV who could not benefit from supratentorial CSF shunt, the traditional valve pressure may not be low enough to drain the CSF from the fourth ventricle, where the CSF pressure may be relatively higher than in the surrounding brain and the rest of the ventricular system. Chari *et al.* reported six cases of patients with DLCFV whose symptoms were resolved by the application of negative CSF pressure systems [5].

Both of our patients were DLCFV. Patient 1 presented with a tetraventricular hydrocephalus that returned to normal after shunting the lateral ventricle, which indicated that the aqueduct had been patent or reopened due to the disappearance of the pressure gradient between the supratentorial and infratentorial compartments. Patient 2 presented with not only tetraventricular hydrocephalus but also syringomyelia and crowded posterior fossa, which fulfilled the concept of isolated rhombencephalic ventricle or holoneural canal dilatation proposed by Qi and Abbott [14]. The dramatic regression of patient 2’s clinical and radiological signs shortly after posterior fossa decompression proved the functional obstruction of the outlets, which was in accord with the definition of DLCFV and prompted the role of the crowded posterior fossa in the pathophysiology of DLCFV.

### Management of DLCFV should be individualized

The management of DLCFV should be individualized. It is critical to differentiate a true symptomatic DLCFV from the other conditions associated with a large fourth ventricle. For DLCFV without clinical abnormality, the surgical intervention may be unnecessary, and careful follow-up is mandatory. Symptomatic DLCFV always requires treatment because of the symptoms induced by the hydrocephalus, the involvement of the brainstem, the cerebellum, or the lower cranial nerves, and the pressure gradient between the posterior fossa and the other compartments. The goal of management is to reestablish normal CSF circulation of the entire ventricular system and subarachnoid space rather than only the fourth ventricle. Zimmerman stated that the dilated fourth ventricle could return to normal following lateral ventricular shunting but that the fourth ventricle must be shunted directly if there is actual obliteration of the aqueduct [17]. Most patients with DLCFV could benefit from supratentorial CSF shunt because the Sylvius aqueduct is patent. However, VP shunt is not always enough to eliminate the pressure gradient between the supratentorial and infratentorial compartments, so there are still some patients with DLCFV who need additional shunting of the fourth ventricle due to the secondary occlusion of the aqueduct. The low-pressure system or negative pressure system via ventriculopleural shunt or valveless VP shunt has also been used to treat DLCFV successfully [5].

Patient 1 was a typical case of DLCFV without crowded posterior cranial fossa or other abnormality. She was cured successfully with a supratentorial VP shunt. However, the decision-making of the treatment strategy of patient 2 has been a puzzle. He had a crowded posterior fossa and cervical syringomyelia, which could have led to the obstruction of the outflow of CSF from the fourth ventricle. Because it has been proved that the DLCFV is not always responsive to VP shunt and the supratentorial CSF shunt, especially the low-pressure shunt system would increase the risk of upward herniation of the cerebellar tissue. Thus, in view of the obviously crowded posterior fossa and the syringomyelia, the posterior fossa decompression with duraplasty turned into the option of initial management. Although the major cerebellar ptosis could not be considered as a good imaging outcome, patient 2 obtained a good clinical outcome. The effectiveness of posterior fossa decompression to treat postshunt IFV has been reported [3]. Our cases suggested that posterior fossa decompression could be a choice for the initial management of the DLCFV coexisting with crowded posterior cranial fossa or syringomyelia.

## Conclusions

DLCFV should be differentiated from IFV. The management of DLCFV should be individualized. If DLCFV coexists with crowded posterior cranial fossa or syringomyelia, the posterior fossa decompression could be an option for initial management. If there is no sign of crowded posterior cranial fossa or syringomyelia, shunt of the lateral ventricles might be the first choice.

## Data Availability

Not applicable.

## Abbreviations

CSF: Cerebrospinal fluid
DLCFV: Disproportionately large communicating fourth ventricle
IFV: Isolated fourth ventricle
MRI: Magnetic resonance imaging
VP: Ventriculoperitoneal
